# Childhood trauma associated with increased post-awakening cortisol in major depressive disorder

**DOI:** 10.1017/S0033291723000053

**Published:** 2023-10

**Authors:** Shabaz Sendi, Susanne Fischer, Andrew Papadopoulos, Lucia Poon, Lena J. Rane, Abebaw Fekadu, Valeria Mondelli, Anthony J. Cleare

**Affiliations:** 1 Department of Psychological Medicine, Centre for Affective Disorders, Institute of Psychiatry, Psychology & Neuroscience, King's College London, London, UK; 2 Affective Disorders Unit and Laboratory, South London and Maudsley NHS Foundation Trust, London, UK; 3 Department of Psychiatry, Austin Health, University of Melbourne, Melbourne, Australia; 4 Institute of Psychology, Clinical Psychology and Psychotherapy, University of Zurich, Zurich, Switzerland; 5 Centre for Innovative Drug Development and Therapeutic Trials for Africa, College of Health Sciences, Addis Ababa University, Addis Ababa, Ethiopia; 6 Global Health & Infection Department, Brighton and Sussex Medical School, Brighton, UK; 7 National Institute for Health Research (NIHR) Mental Health Biomedical Research Centre, South London and Maudsley NHS Foundation Trust and King's College London, London, UK

**Keywords:** Childhood trauma, cortisol, depression, stress, treatment-resistance

## Abstract

**Background:**

Enhanced post-awakening cortisol may serve as a biological marker for individuals with major depressive disorder. However, studies comparing post-awakening cortisol between patients with major depressive disorder (MDD) and healthy controls have produced conflicting findings. The aim of this study was to investigate whether this inconsistency could be due to the effects of childhood trauma.

**Methods:**

A total of *N* = 112 patients with MDD and healthy controls were divided into four groups according to the presence of childhood trauma. Saliva samples were collected at awakening and 15, 30, 45, and 60 min later. The total cortisol output and the cortisol awakening response (CAR) were calculated.

**Results:**

The total post-awakening cortisol output was significantly higher in patients with MDD as compared to healthy controls, but only in those individuals reporting childhood trauma. The four groups did not differ regarding the CAR.

**Conclusions:**

Elevated post-awakening cortisol in MDD may be confined to those with a history of early life stress. Tailoring and/or augmenting of currently available treatments may be required to meet the specific needs of this population.

## Introduction

One of the most promising biological markers of major depressive disorder (MDD) is cortisol, the end product of the hypothalamic-pituitary-adrenal (HPA). Meta-analyses have demonstrated that at least some patients with MDD exhibit elevated basal cortisol and non-suppression of cortisol following the administration of dexamethasone, a synthetic analogue of cortisol (Stetler & Miller, 2011). Further meta-analyses have found that the same pattern predicts non-responses to psychotherapy (Fischer, Strawbridge, Vives, & Cleare, 2017a) and – to some extent – pharmacotherapy (Fischer, Macare, & Cleare, 2017b). These findings underscore the potential of cortisol as a prognostic or prescriptive marker in the treatment of MDD. However, one obstacle to a potential translation of these findings into clinical practice is the fact that the dexamethasone suppression test is invasive and may also involve an overnight stay at a clinic or hospital.

A much less invasive and at the same time highly reliable and ecologically valid alternative to determine cortisol is by means of repeated saliva sampling shortly after awakening (Stalder et al. 2016). This method allows the capture of total cortisol production in the early morning hours as well as the typical rise in cortisol around 30 to 45 min after awakening, the cortisol awakening response (CAR), hence providing a natural measure of both HPA axis activity and reactivity. A number of attempts at delineating post-awakening cortisol in MDD have been undertaken (see Dedovic & Ngiam, 2015 for a review). However, findings are far from conclusive, with both elevated and attenuated levels observed in patients as compared to healthy controls.

One factor that may explain these divergent findings is childhood trauma. It has repeatedly been suggested that the observed HPA axis alterations in MDD are heavily influenced by, and may even be secondary to, the presence of a history of early life stress, such as childhood trauma (Heim, Newport, Mletzko, Miller, & Nemeroff, 2008; Heim, Shugart, Craighead, & Nemeroff, 2010). However, studies that have accounted for a potential influence of childhood trauma in a comparison of post-awakening cortisol between patients with MDD and healthy controls are scarce. One study undertaken in young adults found that the total morning cortisol output was elevated in individuals with childhood trauma when compared to individuals without childhood trauma – regardless of MDD (Lu, Gao, Huang, Li, & Xu, 2016). Furthermore, the CAR was comparably enhanced in healthy individuals with childhood trauma. Another study in older adults confirmed this finding, but did not observe any differences in total cortisol according to diagnostic status or a history of childhood trauma (Wielaard, Schaakxs, Comijs, Stek, & Rhebergen, 2018). Finally, one study in adults did not find any interaction between childhood trauma, depression/anxiety disorders, and total morning cortisol/the CAR (Holleman, Vreeburg, Dekker, & Penninx, 2012).

Given these conflicting findings as well as the general dearth of research in this area, the aim of the present study was to shed further light on the role of childhood trauma in altered HPA axis functioning in MDD by studying individuals across a more clinically representative age range. Based on the literature, it was expected that post-awakening cortisol would be highest in patients with MDD reporting childhood trauma and lowest in healthy controls reporting no childhood trauma, with intermediate values in the two remaining groups.

## Methods

### Participants

Individuals aged 18 to 75 years were recruited into the study. Patients were recruited from inpatient and outpatient services of the Affective Disorders Unit at the Bethlem Royal and Maudsley Hospitals in London, United Kingdom. They underwent full clinical assessment by a psychiatrist and were required to meet both DSM-IV (APA, 2000) and ICD-10 (WHO, 1992) criteria for MDD. Healthy controls with no personal or family history of mental disorders were recruited from the general population, including several volunteers that had previously taken part in other studies of our research group. All were evaluated at the time of data collection. Participants who had any disease that might affect HPA axis functioning, were using corticosteroids, were heavy smokers (more than 40 cigarettes in 24 h), had alcohol or drug dependency, or who were pregnant or nursing were excluded from the study. Patients with MDD were not withdrawn from medication: all were taking an antidepressant and the majority additional augmentation medication as per the Maudsley Prescribing Guidelines. After determining subjects that were suitable to be included, they were then allocated to the appropriate subgroup based upon the presence or absence of a history of childhood trauma (see below). Given the differing prevalence of childhood trauma, the groups were therefore not of equal sizes.

A total of 119 subjects were identified. Of these, five participants were not included because they did not collect the first or last cortisol samples, or because they had two missing values at other time points preventing interpolation of missing data. One participant was not included because of failure to complete sample collection within the appropriate time (collecting over 4 h instead of 60 min). Finally, one participant was not included because the level of cortisol was more than 25 nmol/l at all the time points. The final sample consisted of *N* = 112 participants: *n* = 43 healthy controls without a history of childhood trauma (HC/no CT), *n* = 26 healthy controls with a history of childhood trauma (HC/CT), *n* = 15 patients with MDD without a history of childhood trauma (MDD/no CT), *n* = 28 patients with MDD with a history of childhood trauma (MDD/CT). Although the sample sizes of the four groups were not specifically calculated for the purpose of the present study, the number of recruited individuals is nearly identical to previous research on childhood trauma, depression, and cortisol in young adults (Lu et al. 2016) and was large enough to detect medium-sized effects regarding the group comparisons according to a G*Power analysis (*α* = 0.05, 1−*β* = 0.80).

### Protocol

Participants were assessed either at the Inpatient Affective Disorders Unit (for those who were inpatients at the time of assessment, i.e. 22/28 in the MDD/CT group and 10/15 in the MDD/no CT group) or at the participant's home. For those participants undertaking the study at home, we posted a package consisting of instructions on how to collect saliva, questionnaires and an envelope in order to return the package to the laboratory. Further support and instructions were delivered to them by telephone.

Participants were asked to collect five saliva samples in polypropylene tubes by using the passive drooling method. In case of dry mouth, they were supplied with a piece of parafilm to chew. The sampling was undertaken on any weekday except Monday, with appropriate precautions taken for those working irregular patterns. Women were asked to collect the samples during the follicular phase of their menstrual cycle. Participants were guided by careful instructions to collect five samples of saliva over the course of the morning. The first one was to be collected at awakening and the other samples at 15, 30, 45, and 60 min after the first one. Participants were informed about not brushing their teeth and not eating or drinking at least one hour before collection. In order to control for the time that the subjects gave the samples, participants completed a form for the day they collected the salivary samples. Participants were asked to keep the samples in a fridge and then send them back to the lab the following day. After arriving at the laboratory, samples were kept at −40 °C in the freezer until analysis.

All procedures were approved by the hospital ethics committee. All participants gave written informed consent form prior to participating to the study. The authors assert that all procedures contributing to this work comply with the ethical standards of the relevant national and institutional committees on human experimentation and with the Helsinki Declaration of 1975, as revised in 2008.

### Psychological measures

To assess the severity of their depression, all participants were interviewed using the 21-item version of the Hamilton Depression Rating Scale (HAM-D 21) (Hamilton, 1960). In patients with MDD, information on the age of onset of first depressive episode and the number of previous depressive episodes was additionally collected. The presence or absence of childhood trauma was determined using the Childhood Trauma Questionnaire (CTQ) (Bernstein et al. 2003). The CTQ is a 25-item questionnaire that includes five subscales (emotional abuse, physical abuse, sexual abuse, emotional neglect, and physical neglect). The presence or absence of childhood trauma was evaluated based on validated cut-off scores for moderate to severe trauma.

### Biological measures

Samples were frozen at −40 °C and then cortisol was determined in a small number of sequential batches of approximately 100 aliquots, with all samples from a single subject being analysed in the same batch. Cortisol was determined using the luminescence assay on the ‘Immulite’ (www.diagnostic.siemens.com). The method correlated well with an in-house and previously published time-resolved fluoroimmunoassay (Juruena et al. 2006). It had an analytical sensitivity of 0.2 nmol/l, a mean recovery of cortisol of 106.1% (range from 5 to 65 nmol/l) and inter/intra-assay precision of less than 10% (range 5 to 25 nmol/l).

### Statistical analysis

Post-awakening areas under the curve (AUC) with respect to the ground (AUCg) and increase (AUCi) were calculated to reflect the total post-awakening cortisol output and the CAR, respectively (Pruessner, Kirschbaum, Meinlschmid, & Hellhammer, 2003). Kolmogorov-Smirnov tests were used to determine if the data were normally distributed. Kruskal–Wallis tests, c^2^ tests, and univariate ANOVAs were used to compare the four groups regarding age, sex, Body Mass Index (BMI), depression severity, and childhood trauma severity. Pearson and Spearman correlations as well as c^2^ tests were used to investigate the relationship between age, sex, BMI, and cortisol. Mann–Whitney *U* tests were used to compare the two depressed groups regarding clinical characteristics. Kruskal–Wallis tests including post-hoc tests were calculated to compare the four groups in terms of the AUCg and AUCi. A two-tailed *p* < 0.05 was considered as statistically significant. All data were analysed with SPSS 22.

## Results

### Sociodemographic and clinical characteristics

All sociodemographic and clinical characteristics of the study participants are listed in Table 1. The four groups did not differ regarding their age (*H*(3) = 6.493, *p* = 0.090) or BMI (*F*(3, 88) = 0.791, *p* = 0.502). There was a significant difference in sex distribution (*Χ*^2^ = 8.390, *p* = 0.039), such that the two clinical groups contained more women than the two control groups (*p* = 0.009). However, neither age (*r*_s_ = 0.12, *p* = 0.192; *r*_s_ = −0.05, *p* = 0.632), nor sex (*U* = 1742, *p* = 0.122; *U* = 1644.5, *p* = 0.335), nor the BMI (*r* = −0.11, *p* = 0.311; *r* = −0.12, *p* = 0.255) were associated with the AUCg or AUCi, respectively.

**Table 1.** Sociodemographic and clinical characteristics of the four groups: healthy controls without a history of childhood abuse (HC/no CT), healthy controls with a history of childhood abuse (HC/CT), patients with major depressive disorder without a history of childhood trauma (MDD/no CT), and patients with major depressive disorder with a history of childhood trauma (MDD/CT), Means ± standard deviations are reported in the case of normally distributed data

	HC/no CT (*n* = 43)	HC/CT (*n* = 26)	MDD/no CT (*n* = 15)	MDD/CT (*n* = 28)
Age (years)	48 (22)	47.5 (18.5)	55 (16)	50 (21.8)
Sex (*n*)				
Male	19*	14*	5*	5*
Female	24*	12*	10*	23*
Body Mass Index (kg/m^2^)	25.7 ± 4.5	26.3 ± 5.2	27.4 ± 5.8	27.7 ± 4.2
Depression severity (HAMD-21)	0 (2)***	0.5 (3)***	19 (6)***	24.5 (10.3)***
Age of illness onset (*y*)	–	–	25 (16.3)	29 (16)
Number of depressive episodes	–	–	1 (2)	4 (5)
Childhood trauma (CTQ) score	27 (5)^ƗƗƗ^	48 (18)^ƗƗƗ^	29 (7)^ƗƗƗ^	53.5 (20)^ƗƗƗ^

CTQ, Childhood Trauma Questionnaire; HAMD, Hamilton Rating Scale for Depression (21 item version).

Medians (and interquartile ranges) are reported in the case of non-normally distributed data. Absolute frequencies are reported in the case of categorical data.

* *p* < 0.05 between the four groups, driven by differences between patients with major depressive disorder and healthy controls.

*** *p* < 0.001 in the four post-hoc comparisons involving both patients with major depressive disorder and healthy controls.

ƗƗƗ *p*<0.001 in the four post-hoc comparisons involving both individuals with childhood trauma and without childhood trauma.

The four groups differed in their depression severity (*H*(3) = 82.396, *p* < 0.001), with post-hoc tests revealing that this was due to patients having significantly higher HAMD-21 scores than controls, irrespective of whether childhood trauma was present or not (all relevant comparisons *p* < 0.001). Within the group of patients with MDD, there was no difference between those with *v.* without childhood trauma in terms of the age of illness onset or the overall number of depressive episodes (both *p* > 0.240). The four groups differed significantly regarding the severity of childhood trauma (*H*(3) = 82.942, *p* < 0.001), with post-hoc tests revealing that this was due to traumatised individuals having significantly higher CTQ scores than non-traumatised individuals, irrespective of whether MDD was present or not (all relevant comparisons *p* < 0.001).

### Post-awakening cortisol

**Fig. 1.**
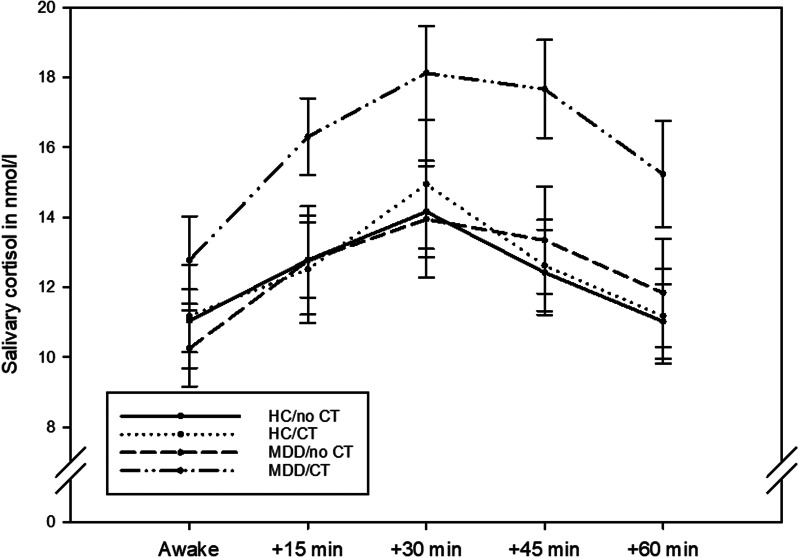
Post-awakening cortisol concentrations in *n* = 43 healthy controls without childhood trauma (CT), *n* = 26 healthy controls with childhood trauma, *n* = 15 patients with major depressive disorder (MDD) without childhood trauma, and *n* = 28 patients with major depressive disorder with childhood trauma. The graph shows mean values and standard errors of the mean.

The four groups did not differ regarding their awakening time (*H*(3) = 1.386, *p* = 0.709). Post-awakening cortisol concentrations are illustrated in Fig. 1. The four groups differed significantly in their AUCg (*H*(3) = 9.569, *p* = 0.023), but not in their AUCi (*H*(3) = 5.882, *p* = 0.117). Post-hoc tests indicated that the significant differences in the AUCg were due to patients with MDD with childhood trauma having a significantly higher AUCg when compared to both healthy controls without childhood trauma (*p* = 0.004) and healthy controls with childhood trauma (*p* = 0.013). The former comparison survived an adjustment for sex (*p* = 0.027) whereas the latter did not (*p* = 0.143).

## Discussion

The present study yielded two findings. First, total post-awakening cortisol production was elevated in MDD when compared to healthy controls, but only among patients who had a history of childhood trauma. Second, the CAR was not found to vary according to diagnostic status or the presence of childhood trauma.

Our first finding is only partially in line with the finding of Lu et al. (2016), who observed an elevated cortisol production in the post-awakening hours in traumatised patients with MDD *and* traumatised healthy controls. However, theirs was a sample of young adults with a mean age of 23 years, whereas the participants of our sample had a mean age of 47 years. It is thus conceivable that, in traumatised individuals who do not develop an MDD, an initial hyperactivity of the HPA axis ‘normalises’ over the course of the lifespan. This could also explain why Wielaard et al. (2018) failed to observe any significant alterations in post-awakening cortisol production in their population-based sample of older individuals with childhood trauma (mean age: 70 years). Notably, in the same study, not even depressed individuals with childhood trauma showed any abnormalities in cortisol output. One way to make sense of this could be that any relationship between MDD or childhood trauma on HPA axis activity in older age is masked by other factors, such as lifestyle behaviours, the presence of physical diseases, or medication intake (Strahler, Skoluda, Kappert, & Nater, 2017). However, given that Holleman et al. (2012), who also used a population-based sample, did not find any effect of childhood trauma on cortisol in middle-aged adults, an alternative explanation could be that elevated post-awakening cortisol is only present in clinical samples of MDD. This would point to the presence of further vulnerability factors in in- and outpatients with MDD, such as genetic/epigenetic markers, which are related to both HPA axis activity as well as inpatient/outpatient status. Multi-level studies, that is, studies incorporating genetic, epigenetic, and endocrine measures, in MDD are warranted to further illuminate this issue.

Our second finding of the CAR being unrelated to the presence of MDD and childhood trauma is only in partial agreement with the findings of Lu et al. (2016) and Wielaard et al. (2018). Whereas both studies confirmed our null-finding regarding MDD, they also observed that healthy individuals with childhood trauma had a significantly greater CAR than all other groups. Whereas the discrepancy with the Lu et al. (2016) study may, again, be understood as a normalisation of HPA activity as traumatised but otherwise healthy individuals age, the discrepancy with the Wielaard et al. (2018) study is more difficult to explain. Again, the Wielaard et al. (2018) study was population-based and hence large enough to enable the investigation of subtypes of childhood trauma. The authors discovered that, in their sample, only emotional and sexual abuse were related to an increased CAR in healthy individuals with childhood trauma. This is in line with a recent meta-analysis, which has demonstrated that only emotional, physical, and sexual abuse were linked with an enhanced CAR (Fogelman & Canli, 2018). A varying distribution of trauma subtypes between our study and others may thus account for the contradicting results. Further, large-scale research is warranted to investigate how different types of childhood trauma may impact the CAR.

Our study presents with a number of strengths. It is the first study in middle-aged adults investigating the role of childhood trauma in post-awakening cortisol of patients with MDD and healthy controls. Furthermore, our sample consisted of well-defined cases with unipolar depression and numerous potential confounders of cortisol were controlled for to maximise the validity of the results. However, a number of limitations equally deserve mention. First, although the overall sample size of this study was reasonably large, the subgroup of depressed patients without childhood trauma was relatively small. Our findings must thus be interpreted with caution and warrant replication in larger samples. Second, our study did not separate diagnostic subtypes of depression. Given that the melancholic subtype in particular has been linked to elevated cortisol (Stetler & Miller, 2011), future large-scale research may be well-advised to include a measure of typicality. Third, although there were some differences in the sex distribution across the four groups, such that the group of individuals with MDD and childhood trauma contained the highest proportion of women. However, it is unlikely that our findings are attributable to this preponderance, since we found no significant link between sex and AUCg/AUCi, and the influence of sex on the CAR has previously been found very small (Fries, Dettenborn, & Kirschbaum, 2009). Fourth, subjects were not medication free, and were taking a variety of antidepressant medications. However, since this caveat applied to both depressed groups, it is unlikely to be the reason why only those with a history of childhood trauma demonstrated increased post-awakening cortisol. Moreover, a large-scale epidemiological study has shown that tricyclic antidepressants appear to reduce rather than to increase post-awakening cortisol levels while SSRIs had no effect on post-awakening cortisol (Manthey et al. 2011) and a clinical study has shown that atypical antipsychotics mildly dampen cortisol (Sarubin et al. 2014). Finally, whilst studies of medication-free individuals may give results unhindered by potential medication related effects, withdrawing treatment from patients in order to undertake studies can itself induce changes due to discontinuation syndromes, may bias sampling to less severely ill populations, and may have ethical implications. Fifth, our post-awakening measurements were confined to one day. Future studies are thus encouraged to use at least two days to increase the reliability of post-awakening cortisol measures (Stalder et al. 2016).

In summary, the present study showed that the total amount of cortisol released after awakening was increased in patients with MDD, although only when they had a history of childhood trauma. This finding resonates well with the notion that at least some neurobiological signatures of MDD may in fact be attributable to early life stress (Heim et al. 2008, 2010). They may also imply that this group of patients is characterised by yet to be determined (epi)genetic alterations, which render them non-resilient to childhood trauma. Our findings are interesting in light of the evidence in favour of polymorphisms in the HPA axis (Fischer, Gardini, Haas, & Cleare, 2019), cortisol (Fischer et al. 2017a, 2017b) as well as childhood trauma (Nanni, Uher, & Danese, 2012) predicting treatment response in MDD. They suggest that there is a biologically distinct subtype of MDD, which may require tailoring and/or augmenting of currently available treatments to meet the specific needs of this population.
